# The effect of abortion on having and achieving aspirational one-year plans

**DOI:** 10.1186/s12905-015-0259-1

**Published:** 2015-11-11

**Authors:** Ushma D. Upadhyay, M. Antonia Biggs, Diana Greene Foster

**Affiliations:** Department of Obstetrics, Gynecology and Reproductive Sciences, Advancing New Standards in Reproductive Health (ANSIRH), Bixby Center for Global Reproductive Health, University of California, San Francisco, 1330 Broadway, Suite 1100, Oakland, CA 94612 USA

**Keywords:** Abortion, Unintended pregnancy, Life goals, Life plans, Aspirations, Outlook, Achievements, Milestones

## Abstract

**Background:**

Women commonly report seeking abortion in order to achieve personal life goals. Few studies have investigated whether an abortion enables women to achieve such goals.

**Methods:**

Data are from the Turnaway Study, a prospective cohort study of women recruited from 30 abortion facilities across the US. The sample included women in one of four groups: Women who presented for abortion just over the facility’s gestational limit, were denied an abortion and went on to parent the child (Parenting Turnaways, *n* = 146) or did not parent (Non-Parenting Turnaways, *n* = 64), those who presented just under the facility’s gestational limit and received an abortion (Near-Limits, *n* = 413) and those who presented in the first trimester and received an abortion (First Trimesters, *n* = 254). Participants were interviewed by telephone one week, six months and one year after they sought an abortion. We used mixed effects logistic regression to assess the relationship between receiving versus being denied abortion and having an aspirational one year goal and achieving it.

**Results:**

The 757 participants in this analysis reported a total of 1,304 one-year plans. The most common one-year plans were related to education (21.3 %), employment (18.9 %), other (16.3 %), and change in residence (10.4 %). Most goals (80 %) were aspirational, defined as a positive plan for the next year. First Trimesters and Near-Limits were over 6 times as likely as Parenting Turnaways to report aspirational one-year plans [Adjusted Odds Ratio (AOR) = 6.37 and 6.56 respectively, *p* < 0.001 for both]. Among all plans in which achievement was measurable (*n* = 1,024, 87 %), Near-Limits (45.6 %, AOR = 1.91, *p* = 0.003) and Non-Parenting Turnaways (47.9 %, AOR = 2.09, *p* = 0.026) were more likely to have both an aspirational plan and to have achieved it than Parenting Turnaways (30.4 %).

**Conclusions:**

These findings suggest that ensuring women can have a wanted abortion enables them to maintain a positive future outlook and achieve their aspirational life plans.

## Background

Women report having abortions for a variety of reasons related to achieving personal life goals. A recent national study based on data from the Turnaway study (which is also the data source for the current study), found that among the primary reasons for wanting an abortion were: feeling not financially prepared (40 %), not the right time (36 %), and having a baby now would interfere with future opportunities (20 %) [1]. Another national study conducted in 2004 among 1209 abortion patients found that the primary reasons for abortion are to mitigate the effects of unintended pregnancy on life course plans [2]. Specifically, among the top reasons women reported having an abortion were: a baby would dramatically change their lives, that they could not afford a baby now, that they did not want to be a single mother or had problems with their relationship, and that they were not ready for a child or another child. Many of these reasons suggest that women felt that carrying the unintended pregnancy to term would interfere with their plans and that abortion would help them achieve their personal goals.

Kirkman and colleagues reviewed the literature on reasons women have abortions. Of the 19 papers they reviewed that met the inclusion criteria, they found that almost all papers included reasons that are classifiable as wrong timing, “which encompassed a sense of not being ready for motherhood and the desire not to disrupt education, work, or life plans”[3].

Several legal scholars and philosophers have used a gender equality framework to support abortion and reproductive rights [4, 5]. The gender equality framework contends that the right to abortion is necessary to ensure equality between men and women. Alison Jaggar argues, “The social assignments of caretaking and often financial responsibility for their children to mothers means that the birth of a child, especially an unwanted child, often severely disrupts women’s life plans” [6].

Popular support for abortion is often based on a desire for women to have access to life opportunities [7]. A recent poll conducted in two states in the US found that the public considers motherhood or being a primary caregiver as one of the top “things [that] might prevent women from having the same opportunities in life or in work as men.”

Despite the prevalent attitudes that abortion enables women to pursue life’s opportunities, only a couple of studies have investigated whether an abortion enables one to achieve specific milestones, and such studies usually focus on educational achievements. For example, a 2-year longitudinal U.S. study found that black teenagers from Baltimore who had an abortion were more likely to continue their education than those who carried to term or those who had never been pregnant [8]. Similarly, a 25-year longitudinal study in New Zealand examined the extent to which abortion mitigated educational, economic, and social disadvantages associated with pregnancy among women less than age 21 [9]. The study found that compared to young women who had unintended pregnancies and carried to term and young women who did not have unintended pregnancies, young women who obtained abortions were more likely to achieve educational milestones. However, there were no differences found in achievement of economic or relationship milestones. The study also found that family, social, and educational characteristics were more likely to explain subsequent life outcomes than whether the woman had an abortion.

Both of these studies had a narrow focus—they looked at adolescent women and used predetermined goals such as high school graduation. They did not include women across the lifespan nor did they consider the woman’s own stated life goals. The one U.S. study was done in a single city (Baltimore), and published over two decades ago when access to abortion services and economic conditions were different. Therefore, findings from that study may not be generalizable to the current U.S. context as a whole.

Probably the greatest weakness of these studies, is that they did not include appropriate comparison groups. Women choosing to have an abortion after an unintended pregnancy may be systematically different than those who never had an unintended pregnancy or those who chose to carry to term. Such unobserved factors may confound any effects found between choosing abortion and achieving life milestones. This study overcomes these methodological weaknesses by comparing two groups of women seeking abortion; women obtaining a wanted abortion compared to women denied a wanted abortion.

Data from University of California, San Francisco’s Turnaway Study were used to examine the impact of having an abortion on women’s own reported one-year plans. Women who obtained a wanted abortion were compared to women who wanted an abortion but were turned away from getting the procedure because they presented for care after the provider’s gestational limit. First, all one-year plans were categorized and it was determined whether each plan expressed a positive goal for the coming year (aspirational). It was assessed whether women who were able to have a wanted abortion were more likely to report an aspirational one-year plan than women denied an abortion. Second, it was assessed whether women who were able to have a wanted abortion were more likely to achieve these aspirational one-year plans one year later.

## Methods

The Turnaway Study is a 5-year longitudinal study of women seeking abortion. The study was designed to assess a variety of outcomes of receiving an abortion compared with carrying an unwanted pregnancy to term. The study received approval from the University of California, San Francisco, Committee on Human Research. All participants provided informed consent.

From 2008 to 2010, the Turnaway Study recruited women from 30 abortion facilities across the United States. Study sites were identified using the National Abortion Federation membership directory and by referral. Sites were selected based on their gestational age limits to perform an abortion procedure, where each facility had the latest gestational limit of any facility within 150 miles. Gestational age limits ranged from 10 weeks to the end of the second trimester. Facilities performed over 2,000 abortions a year on average [10]. They were located in 21 states distributed relatively evenly across the country.

Women were recruited on a 1:2:1 ratio: women who presented up to 3 weeks over the facility’s gestational age limit and were turned away (“Turnaways”), women who presented up to 2 weeks under the limit and received abortions (“Near-Limits”), and women who presented in the first trimester and received abortions (“First Trimesters”). Since the majority (92 %) of abortions in the U.S. occur in the first trimester of pregnancy [11], comparisons between the Turnaways and the First Trimesters served to assess whether the experiences of women seeking later abortions differ from the typical experience of women having abortions in the U.S.

It was anticipated that relatively few women would meet the Turnaway eligibility requirements; therefore, to ensure a large enough overall sample for analysis without being restricted by the low number of women eligible for the Turnaway group, twice as many Near‐Limit participants were enrolled as Turnaways or First‐Trimester participants. For this analysis, the Turnaway group was divided into Parenting Turnaways and Non-Parenting Turnaways (which included Turnaways who subsequently had an abortion elsewhere, reported that they had miscarried, or placed the child for adoption).

Women were eligible for participation if they sought an abortion within the gestational limits for each of the study groups, spoke English or Spanish, and were aged 15 years or older. Further details on recruitment and methods can be found elsewhere [12, 13]. After the baseline survey, participants were contacted for a follow-up phone interview every six months for five years. Turnaway Study data for this analysis come from interviews done at baseline (one week), six months, and one year after they were recruited at their abortion-seeking visit.

To reduce losses to follow up, researchers collected detailed contact information and participants’ preferred methods of communication and confidentiality protection preferences; they also called women after two months to confirm that the woman’s primary and secondary contact information was still valid. When participants could not be reached, researchers called each day for up to 5 days. If she still could not be reached, researchers sent up to 3 follow-up letters by mail or email (according to her stated contact preferences) and continued to call at the same frequency for a maximum of 10 sequential days. To compensate respondents for their time, each received a $50 gift card to a large retail store upon completion of each interview.

### Measures

During the baseline Turnaway Study interview, participants were asked about sociodemographic characteristics, their reproductive histories, and a final, open-ended question “How do you think your life will be different a year from now?” which was used to capture respondents’ one-year plans. Respondents were permitted to provide as long a response as desired. The 6-month and one-year follow-up interviews included questions about whether they were going to school, whether they were working full or part time, what they did for work, their personal and household income, their household composition, their relationships, their children, their life satisfaction, and their emotions regarding the abortion. These items were used to assess whether women achieved their one-year plans.

Many women reported multiple one-year plans. Each individual plan in a dataset that was blinded to study group was considered (although some women’s plans were suggestive of her study group). Each plan was categorized by topic: Education, Employment, Financial, Child-related, Emotional, Living Situation/Residence, Relationship Status, and Other. The Other category included vague plans, plans for personal growth, car ownership, health and other plans that did not fit into one of the other eight topics.

Then, the outlook of the plan was determined—whether it was positive, negative or neutral. This determination was based on the tone of the statement and the qualifiers used. If determination was unclear, the plan was categorized as neutral. Two researchers reviewed each plan. Identification of a plan as positive or negative required both researchers agreeing. Positive plans are referred to as “aspirational.”

Finally, survey items in the six-month and one-year interviews that would indicate achievement of the plan were identified. Some specific plans required all co-authors to discuss and agree upon the meaning of the plan and whether our interview items were sufficient to measure achievement. The exact timing for residential moves could not be determined so when a plan involved a residential move, she was considered to have achieved the goal if there was evidence that she moved by the second year of the study.

### Data analysis

First, sample was described, comparing the socio-demographic characteristics of each group to the Turnaway-Parenting group. For all analyses, mixed-effects regression models that included random effects for facility were used, and *p*-values that adjust for the clustering of participants within each site are presented. The Turnaway-Parenting group was the reference category for all comparisons.

One-year plans were described by topic and by outlook (negative/neutral/positive). Mixed-effects multinomial logistic regression was used to assess differences in proportions among the study groups.

Finally, two mixed-effects logistic regression models were conducted: The first modeled the likelihood of having an aspirational one-year goal and the second modeled the likelihood of having an aspirational goal and achieving it. Both models assessed the effects of study group and adjusted for baseline covariates: age, race, education, employment, poverty status, union status, parity, and history of anxiety/depression. The unit of analysis was one-year plans and because some women reported multiple plans, mixed-effects models were used to account for clustering by woman and within each site. Statistical significance was set at *p* < 0.05 for all comparisons and adjusted odds ratios (AORs), and 95 % confidence intervals are reported. All statistical analyses were performed using STATA 13 (Stata Corp, 2012).

## Results

**Fig. 1:**
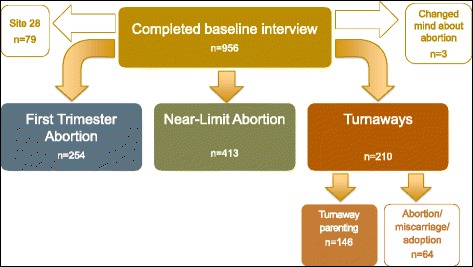
Sample by study group

Overall, 37.5 % of eligible women consented to complete semi-annual telephone interviews for five years, with no differential participation by study group. A total of 956 women completed a baseline interview 8 days after seeking an abortion. One facility was excluded (*n* = 76) from all analyses because 95 % of women initially denied an abortion obtained one elsewhere, and thus the site did not contribute an adequate sample of Turnaways. Three women in the Near-Limit abortion group and First-Trimester group were excluded because they reported that they chose not to have an abortion after agreeing to participate in the study, leaving a final sample of 877 participants at baseline. This analysis was limited to those who completed a one-year follow up interview—146 Parenting Turnaways, 254 First-Trimesters, 413 Near-Limits, and 64 Non-Parenting Turnaways (see Fig. 1). Of the 877 participants who completed the first interview, 86 % also completed the one year follow-up interview with no differences between those with follow-up data and those who were lost to follow up in the kinds of plans reported at baseline. The final sample of participants in this analysis was 757.

### Participant characteristics

The only significant differences in socio-demographic characteristics between the Near-Limit Abortion group and the Parenting Turnaway group (among those with one year follow up data) were age and parity (see Table 1). Parenting Turnaways were younger and less likely to have previous children than Near-Limits. They did not differ significantly by race, education, marital status, school/employment status, history of child sexual abuse, or history of anxiety or depression.

### Topics of one-year plans

Because each respondent could give multiple one-year plans, the 757 respondents reported a total of 1,304 plans. Among all participants, plans were distributed among the following themes: Educational (21.3 %), Employment (18.9 %), Other (16.3 %), Changes in Living Situation/Residence (10.4 %), Child-related (10.3 %), Financial (7.8 %), Relationship (5.3 %), Emotional (5.1 %), and Don’t know (4.5 %).

At baseline, approximately one week after receiving or being denied an abortion, women in the Parenting Turnaway group were most likely to mention one-year plans related to children—significantly more than Near-Limits, First Trimesters (both *p* < 0.001), and Non-Parenting Turnaways (*p* = 0.001).

**Fig. 2:**
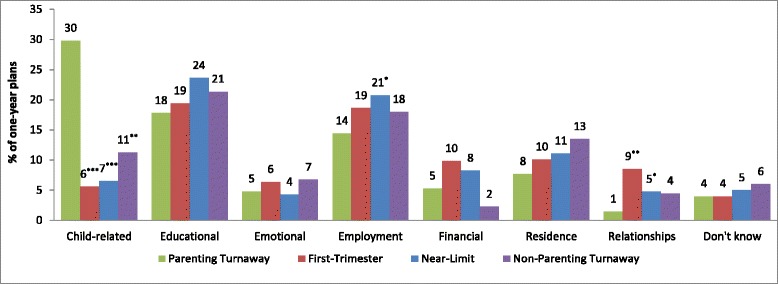
Proportion of one-year plans by topic/theme category, by study group, *n* = 1,304 plans. % of one year plans is significantly different than Parenting Turnaways at *p < 0.05, **p < 0.01, or ***p < 0.001

Parenting Turnaways were significantly less likely to mention one-year plans related to employment than Near-Limits (*p* = 0.045). They were also significantly less likely to mention one-year plans related to relationships than Near-Limits (*p* < 0.045) and First Trimesters (*p* < 0.002) (see Fig. 2).

### Outlook of one-year plans

The majority of one-year plans were aspirational (80.2 %), followed by neutral/matter of fact one-year plans (17. 6 %) and negative one-year plans (2.2 %). The following are examples of typical aspirational one-year plans in each category (each quoted clause represents a different participant):

Child-related: “Give a good life to my kids,” “My daughter will be done with the first year of high school.”

Education: “I hope that I will be back in school,” “Finished my education.”

Emotional: “I just want to be happy,” “Less stressful.”

Employment: “have a better job,” “Hopefully I’ll be opening my own business.”

Financial: “more financially stable,” “more money,” “I am hoping to be able to support me and my daughter on my own.”

Residence: “won’t live with my parents anymore,” “I’ll probably be in a different country, hopefully Australia,” “have my own place for me and my son.”

Relationships: “I’ll be married,” “I hope to be divorced,” “better relationship,” “As long as I stay away from the person I was with, I’ll be 100 % better.”

Other: “I’m hoping to take better care of myself,” “Have my own car,” “Good, I mean, I don’t know.”

Neutral/matter of fact responses most often included having a child, but also included statements about life being the same, or life being different without further comment suggesting how the respondent felt about it. The following are examples of typical neutral one-year plans in each category:

Child-related: “I guess I will have three children instead of two,” “Kids will be older.”

Emotional: “This experience has changed me. I can’t quite articulate it yet but I imagine it will still be impacting me a year from now”

Residence: “In process of moving.” “living situation will be the same.”

Relationships: “I don’t plan on having a family or getting married.” “I don’t think I want to have any relationships. Or think about anything like that”

Other: “I don’t know,” “I don’t think it will be any different.”

Among all groups, there were 30 negative one-year expectations and one-third of these focused on the change in quality of life and the woman’s emotions with a new child. The following are examples of typical negative one-year plans in each category:

Child-related: “More stressful and hectic with having two kids” and “I’ll be running back and forth to day care having to pay someone to watch my child.”

Education: “I don’t think I’ll be going to school,” “I am going to have to work twice as hard to get through school and stuff.”

Emotional: “I’ll still be thinking about the abortion,” “It will be very different. I don’t think I will be happy. It will be very difficult for me. I don’t know what I will do.”

Employment: “I believe that I will be working two jobs, working really hard to support two kids.”

Financial: “I think that I will have four children instead of three and I will probably have less money,” “My living situation is all I can afford.”

Residence: “I won’t be living with my family and I’ll have a kid. I think it will be a little bit more challenging.”

Other:” I’m living day by day, so I don’t know.” “I think that it will be the same. I don’t see a future.”

**Fig. 3:**
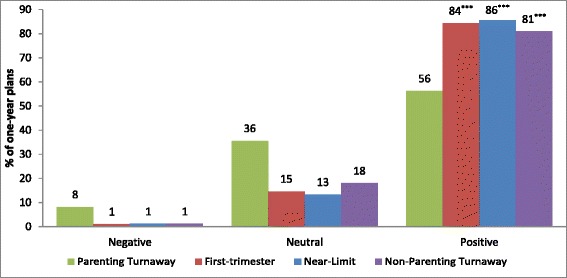
Proportion of one-year plans by whether they were negative, neutral/matter of fact or positive, by study group, *n* = 1,304. ***% of one year plans is significantly different than Parenting Turnaways at *p* < 0.001

One-year plans were significantly more likely to be aspirational among First Trimester (84.3 %), Near-Limit (85.6 %), and Turnaway-Not Parenting (80.9 %) groups compared to the Turnaway-Parenting group (56.3 %, *p* < 0.001 for all comparisons) (see Fig. 3). In a model adjusting for potential covariates, First Trimesters and Near-Limits were over 6 times as likely as Parenting Turnaways to report aspirational one-year plans (Adjusted Odds Ratio (AOR) = 6.37 and 6.56 respectively, *p* < 0.001 for both). Non-Parenting Turnaways were four times as likely to report aspirational one-year plans (AOR = 4.00, *p* < 0.001). The only other significant predictor of having an aspirational plan was marital status with married women less likely to have positive one-year plans than unmarried women (70.9 % vs 81.1 %, AOR = 0.56, *p* = 0.04) (see Table 3).

**Table 1:** Baseline characteristics of sample and distribution by study group (*n* = 757)

	Parenting turnaways (PT)	First trimesters (F)	Near limits (N)	Non-parenting turnaways (NPT)	Total	N	*p*-value	*p*-value	*p*-value
	(*n* = 146)	(*n* = 254)	(*n* = 413)	(*n* = 64)			PT vs F	PT vs. N	PT vs. NPT
Total	16.2	29.9	46.9	7.0	100.0	757			
Age category									
15–19^a^	29.3	14.2	16.1	18.9	17.8	135			
20–24	33.3	27.9	40.0	43.4	35.5	269			
25–34	33.3	49.1	36.6	30.2	39.4	298			
35–46	4.1	8.8	7.3	7.5	7.3	55	0.001	0.02	NS
Race									
Non-Hispanic white	23.6	39.8	31.8	37.7	33.3	252			
Non-Hispanic black	35.8	31.9	32.7	32.1	32.9	249			
Latina	26.8	20.4	21.7	20.8	22.1	167			
Multiracial/other	13.8	8.0	13.8	9.4	11.8	89	0.02	NS	NS
Highest level of education									
Less than HS	22.8	15.9	17.7	15.1	17.8	135			
HS or GED	32.5	28.3	34.6	32.1	32.2	244			
AA, some college, tech school	38.2	44.7	40.6	45.3	41.7	316			
College degree	6.5	11.1	7.0	7.5	8.2	62	NS	NS	NS
Marital status									
Not married	88.6	88.1	92.1	96.2	90.6	686			
Married	11.4	11.9	7.9	3.8	9.4	71	NS	NS	NS
Employment									
Not in school or employed	33.3	17.7	24.8	24.5	24.0	182			
In school or employed (or homemaker)	66.7	82.3	75.2	75.5	76.0	575	0.05	NS	NS
Gestation at Baseline									
3–13 weeks	1.4	99.7	13.9	0	35.7	466			
14–19 weeks	19.2	0.3	19.3	49.4	15.9	207			
20+ weeks	79.3	0	66.7	50.6	48.4	631	NA	NS	NA
Number of children									
None	49.6	40.9	37.0	41.5	40.5	306			
1	21.1	23.6	30.5	28.3	26.8	202			
2 or more	29.3	35.6	32.5	30.2	32.7	247	NS	0.04	NS
History of child sexual abuse or neglect									
No	74.8	72.6	73.2	79.2	73.7	558			
Yes	25.2	27.4	26.8	20.8	26.3	199	NS	NS	NS
History of diagnosed anxiety or depression									
No	69.8	69.0	76.6	69.8	74.5	564			
Yes	30.2	31.0	23.4	30.2	25.5	193	0.04	NS	NS

^a^This age category includes one participant aged 14 who was recruited early in the study before the minimum enrollment age was changed to 15

*NS* Not significant, *NA* Not applicable

*P*-value could not be computed because of empty cell

**Table 2:** Adjusted odds of a having an aspirational one-year plan and adjusted odds having an aspirational one-year plan and achieving it

	Adjusted odds of having an aspirational one-year plan		Adjusted odds of having an aspirational one-year plan and achieving it	
	(*n* = 1,304)		(*n* = 1,024^a^)	
	Adj. odds ratio	95 % CI	Adj. odds ratio	95 % CI
Study Group				
Parenting Turnaway	ref		ref	
First trimester	6.37***	(3.53,11.49)	1.57	(0.98,2.50)
Near-Limit	6.56***	(3.80,11.32)	1.91**	(1.25,2.93)
Turnaway-Non Parenting	4.00***	(1.89,8.50)	2.09*	(1.09,3.99)
Sociodemographic characteristics				
Age category				
14–19	1.94	(0.84,4.48)	0.77	(0.39,1.52)
20–24	1.23	(0.60,2.51)	0.77	(0.42,1.43)
25–34	1.04	(0.52,2.08)	0.90	(0.49,1.63)
35–46	ref		ref	
Race/ethnicity				
Non-Hispanic White	ref		ref	
Non-Hispanic Black	0.97	(0.62,1.51)	0.84	(0.58,1.20)
Hispanic/Latina	0.90	(0.56,1.46)	0.74	(0.49,1.10)
Multiracial/other	1.07	(0.60,1.93)	0.82	(0.52,1.31)
Highest grade completed				
Less than high school	0.86	(0.52,1.44)	0.77	(0.51,1.18)
High school diploma or GED	ref		ref	
Some college, vocational training, or Associates degree	0.91	(0.60,1.37)	1.06	(0.76,1.49)
College degree	1.01	(0.49,2.08)	0.90	(0.51,1.62)
Marital status				
Married	0.56*	(0.32,0.98)	0.80	(0.47,1.35)
Not married	ref		ref	
Employment				
Not in school or employed	ref		ref	
In school or employed (or homemaker)	0.89	(0.59,1.34)	0.85	(0.60,1.19)
Previous children				
0	ref		ref	
1	0.77	(0.50,1.19)	0.72	(0.51,1.03)
2+	0.97	(0.59,1.57)	0.70	(0.47,1.04)
History of child sexual abuse				
No	ref		ref	
Yes	0.96	(0.64,1.44)	1.23	(0.87,1.73)
History of diagnosed anxiety or depression				
No	ref		ref	
Yes	1.08	(0.71,1.64)	0.88	(0.62,1.25)

^a^Excludes plans that are not measurable

*p <0.05, **p <0.01, or ***p <0.001

**Table 3:** Total number of aspirational plans that were unmeasurable, measurable and percentage of measurable plans that were achieved

Goal type	Unmeasurable	Measurable	% of measurable plans that were achieved
	N	N	
Child-Related	10	18	88.9
Financial	0	96	72.9
Other	83	69	72.5
Residence	4	122	59.8
Employment	2	237	44.7
Emotional	6	50	40.8
Educational	4	269	30.9
Relationship status	24	39	18.0
Total	133	900	47.3

### Achievement of one-year plans

Among the 1,046 total aspirational plans across study groups, it was possible to assess whether 87.1 % were achieved by one year using a range of items included in the interview guide. The most common measures used to assess achievement of plans included whether the participant obtained a specific degree or graduated, whether she had a higher income, whether she was in school, whether she was working, whether she moved out of her parents’ house and/or living out on her own, whether she moved, and whether she felt satisfied with her life (used to evaluate happiness).

Achievement of 12.9 % (*n* = 133) of life plans could not be measured because they were either too vague or appropriate data to verify if the goal was achieved was unavailable. For example, vague unmeasurable goals included: “I hope and think I’m going to be more on track—more stable. Getting everything straightened up” and “Hopefully be in a better more stable place.” Wanting greater stability in the future was a common unmeasurable theme. Goals that were unmeasurable also included those for which no information was collected such as goals about car ownership, being in a good relationship with a new partner, and participants’ hopes for family members’ achievements.

Among the 899 aspirational plans that were measurable, 47.3 % were achieved. There was no difference by study group in the achievement of aspirational plans among women who reported them—Parenting Turnaways: 46.2 %, First Trimesters: 44.7 %, Near-Limits: 48.3 %, the Non-Parenting Turnaways: 52.3 % (not shown in tables). Among the measurable aspirational plans, women were most likely to achieve child-related plans (88.9 %), which most often entailed having a new baby. Women were also highly likely to achieve their financial (72.9 %) and other plans (72.5) within one year. They were least likely to achieve their educational (30.9 %) and relationship status (18.0 %) plans (Table 2). There were no significant differences in achievement within each plan type by study group.

However, among all measurable plans (*n* = 1,024), Near-Limits (45.6 %, AOR = 1.91, *p* = 0.003) and Non-Parenting Turnaways (47.9 %, AOR = 2.09, *p* = 0.026) were significantly more likely to have both an aspirational plan and to have achieved it than Parenting Turnaways (30.4 %) (see Table 3).

## Discussion

This study found that women who were denied an abortion were less likely to have aspirational one-year plans than those who obtained an abortion. Those who were denied an abortion were more likely to have neutral or negative expectations for their future. Whether or not a person has aspirational plans is indicative of her hope for the future. Without such plans or hopes, she misses out on opportunities to achieve milestones in life.

These findings suggest that shortly after being denied an abortion, many Turnaways may have scaled back their one year plans knowing that they were going to have to carry an unwanted pregnancy to term. Turnaways likely changed their one year plans in two ways after learning of being denied an abortion: First, they often incorporated their forthcoming child into their aspirational one-year plans; these child-related goals were often achieved simply by carrying the pregnancy to term. Turnaways were significantly less likely to have vocational goals compared to women who obtained an abortion, likely because employment-related goals felt unattainable while parenting a newborn. Second, women who were denied a wanted abortion were adjusting to the idea of carrying an unwanted pregnancy to term and likely changed from having more aspirational one-year plans to more neutral or negative expectations for the future.

The greater focus on relationship goals among women in the Near-Limit group may reflect their desires for new and better relationships; women who have an abortion may feel free to leave poor relationships compared to women who are going to have a child with the man involved in the pregnancy. Indeed, as reported in other papers from these data, one-third of participants reported their partner as a reason to have an abortion, including poor relationships and undesirable characteristics for fatherhood [14] and women denied an abortion were slower to end a relationship with the man involved in the pregnancy compared to Near-Limits who received their wanted abortion [15].

In addition to the straightforward goals of gaining employment or education, many women mentioned personal psychosocial goals they wanted to achieve. A strength of this study is that many points of data on a wide variety of psychosocial and emotional outcomes were available, including life satisfaction, anxiety, and depression allowing us to assess achievement in goals related to mood and happiness which were relatively common. One construct that was not measureable was stability, a common theme among women’s visions for the future. Future studies should aim to measure life stability as well as other emotional outcomes to understand how they are affected by pregnancy decisions.

A strength of the study was the use of appropriate comparison groups to understand the effects of abortion. All of the women in our sample had unintended pregnancies and all sought abortion. Comparing those who were denied an abortion to those who received a wanted abortion allows us to control for any unobserved characteristics that would be associated with abortion-seeking for example, the life circumstances that brought women to their abortion decision. In addition, confounders thought to affect our outcome measures were controlled for.

While most women in all groups had positive one-year plans, fewer than half of the goals were achieved within one year. In other words, many women overestimated what they could achieve in one year.

This study has several limitations. First, the Turnaway study is limited to fewer than one thousand women and many women who were invited to participate declined. This study’s participation rate is in line with other longitudinal studies [16, 17] yet the women who declined to participate may be different from those who agreed. This analysis enjoyed a relatively high one-year follow-up rate (86 %) with no differentials in the kinds of plans reported by those who completed the one-year interview and those who did not. Additionally, due to sample size limitations, the analysis was unable to determine achievement by specific theme of the goal. Another limitation is that the analysis was unable to evaluate whether all goals were met and for some goals, measurement may have been imprecise, for example, the timing of residential moves. Finally, because many Turnaways likely changed their goals after learning they were denied an abortion, it could not be determined how abortion (or being denied an abortion) affected the women’s original goals, before some learned they were going to have to carry to term. Future studies should attempt to assess personal goals before unintended pregnancy to further understand the effect of abortion on life course outcomes.

## Conclusion

This study demonstrates that women who receive a wanted abortion are better able to aspire for the future than women who are denied a wanted abortion and must carry an unwanted pregnancy to term. Support for a woman to have access to abortion is often based on a belief that when faced with an unintended pregnancy, women who have an abortion have better life course trajectories than women who carry their unintended pregnancies to term. There is a belief that access to abortion is important for equal opportunities for women and for their financial stability [7]. These findings provide evidence to support this premise.

Women seek abortion for a range of reasons tied to their individual life circumstances and stage of life and oftentimes for the profound effects they perceive that having a baby would have on their life plans. Our analysis is unique because it allowed women to express their life plan in their own words. This study shows that abortion enables women to aspire for a better life in the future and achieve these goals.
